# Host Defense Peptides at the Ocular Surface: Roles in Health and Major Diseases, and Therapeutic Potentials

**DOI:** 10.3389/fmed.2022.835843

**Published:** 2022-06-16

**Authors:** Darren Shu Jeng Ting, Imran Mohammed, Rajamani Lakshminarayanan, Roger W. Beuerman, Harminder S. Dua

**Affiliations:** ^1^Academic Ophthalmology, School of Medicine, University of Nottingham, Nottingham, United Kingdom; ^2^Department of Ophthalmology, Queen's Medical Centre, Nottingham, United Kingdom; ^3^Anti-Infectives Research Group, Singapore Eye Research Institute, Singapore, Singapore

**Keywords:** antimicrobial peptide, cathelicidin, defensin, dry eye, host defense peptide, infection, keratitis, ocular surface

## Abstract

Sight is arguably the most important sense in human. Being constantly exposed to the environmental stress, irritants and pathogens, the ocular surface – a specialized functional and anatomical unit composed of tear film, conjunctival and corneal epithelium, lacrimal glands, meibomian glands, and nasolacrimal drainage apparatus – serves as a crucial front-line defense of the eye. Host defense peptides (HDPs), also known as antimicrobial peptides, are evolutionarily conserved molecular components of innate immunity that are found in all classes of life. Since the first discovery of lysozyme in 1922, a wide range of HDPs have been identified at the ocular surface. In addition to their antimicrobial activity, HDPs are increasingly recognized for their wide array of biological functions, including anti-biofilm, immunomodulation, wound healing, and anti-cancer properties. In this review, we provide an updated review on: (1) spectrum and expression of HDPs at the ocular surface; (2) participation of HDPs in ocular surface diseases/conditions such as infectious keratitis, conjunctivitis, dry eye disease, keratoconus, allergic eye disease, rosacea keratitis, and post-ocular surgery; (3) HDPs that are currently in the development pipeline for treatment of ocular diseases and infections; and (4) future potential of HDP-based clinical pharmacotherapy for ocular diseases.

## Introduction

The ocular surface (OS) is a specialized anatomical and functional system composed of various structures and components, including the tear film, conjunctival and corneal epithelium, lacrimal glands, meibomian glands, and nasolacrimal drainage apparatus. Originating embryologically from the surface ectoderm, all these OS structures are linked anatomically *via* the epithelium and functionally *via* the regulation of neuronal, vascular, endocrinological, and immunological systems (1). Together, they maintain the homeostasis of the OS which has critical roles in the optical quality of the eye to focus light at the retina and serving as the most front-line defense system of the eye against a wide array of pathogens as well as physical and chemical insults (2). In addition, the periocular skin, which is in close vicinity to the eye, has important influences on the health of OS. Inflammatory diseases of the periocular skin such as atopic dermatitis and rosacea often result in the manifestation of OS damage (3–5).

Being constantly exposed to pathogens, environmental irritants and stress, the OS relies on a highly functional innate immunity. Innate immunity mechanisms for the OS are composed of three major components, including the physical barrier (e.g., epithelial layers of conjunctiva and cornea), chemical barriers (e.g., tears), and cellular responses (e.g., macrophages, neutrophils, and others), for which the host defense peptides (HDPs) play important roles in the latter two.

**Figure 1:**
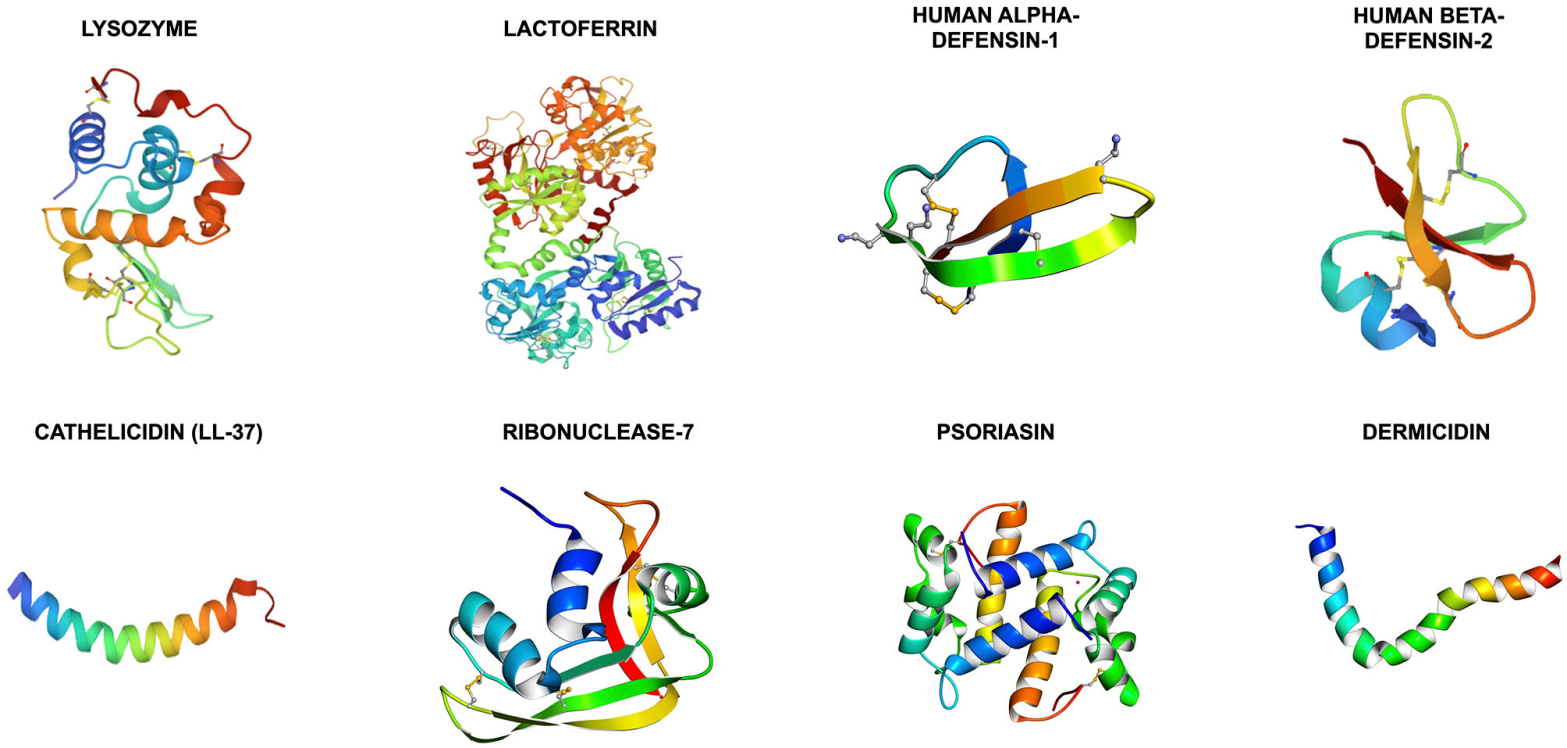
Illustrations of the 3-dimensional secondary structures of the important host defense peptides (HDPs) at the ocular surface. The structures are obtained from the RCSB Protein Data Bank. Cathelicidin, psoriasin, and dermcidin are primarily made of alpha-helical helixes whereas human alpha- and beta-defensins are composed of triple beta-sheets. Lysozyme, lactoferrin and ribonuclease-7 (or RNase-7) are made of mixed alpha-helixes and beta-sheets.

**Figure 2:**
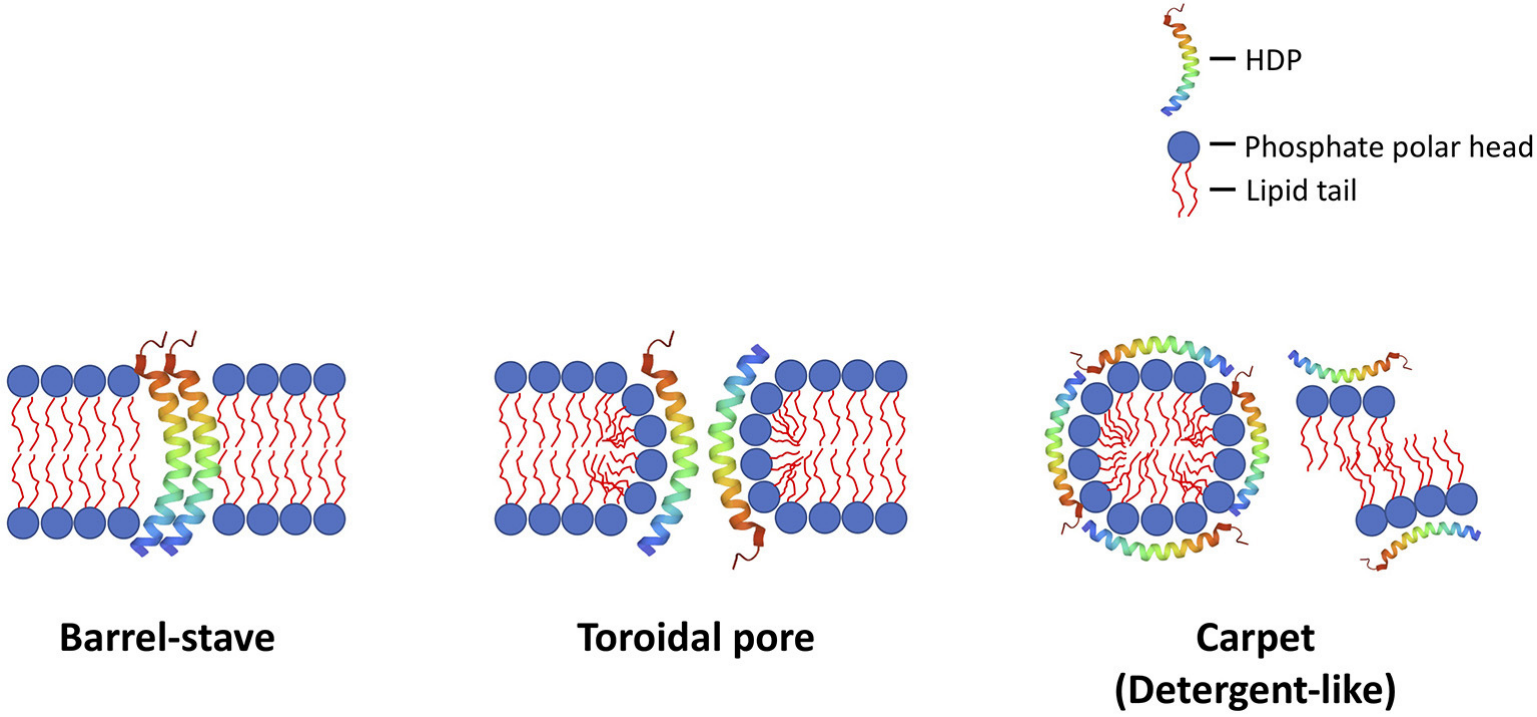
Illustrations of the common membrane-permeabilizing mechanisms of host defense peptides (HDPs) against bacteria (and other microbes), namely: (1) barrel-stave; (2) toroidal pore; and (3) carpet (detergent-like) mechanisms. In the barrel-stave model, the HDPs act as a stave and penetrate vertically into the negatively charged, lipid bilayer bacterial membrane, creating permanent “barrel-shaped” pores. In the toroidal pore model, the HDPs interact with the negatively charged phosphate head groups electrostatically, distort the arrangement of the lipid bilayer, and create a transient membrane pore, with HDPs lining and stabilizing the internal part of the pore. In the carpet (detergent-like) model, the HDPs interact with the bacterial membrane electrostatically, and, upon reaching the critical concentration on the bacterial membrane, they result in membrane fragmentation / aggregation. These mechanisms result in destabilization of the membrane integrity, which leads to influx of fluid and efflux of intracellular content, culminating in cell lysis. Although less common (not shown in this figure), HDPs may also exert their antimicrobial action *via* binding to microbial intracellular targets (i.e., non-membrane-permeabilizing mechanisms), inhibiting DNA/RNA synthesis, protein synthesis and protein folding).

Antimicrobial peptides (AMPs) are a group of evolutionarily conserved molecules of the innate immunity (6). To better capture the increasingly recognized multi-faceted roles of AMPs, a broader term “host defense peptides (HDPs)” has been subsequently introduced (7). They are ubiquitously expressed at epithelial surfaces (e.g., eye, skin, respiratory, gastrointestinal linings, etc.) and secreted by immune cells (e.g., polymorphonuclear leukocytes and macrophages) (8, 9). So far more than 3,000 naturally occurring and synthetic HDPs have been discovered across six life kingdoms (10, 11). These HDPs are usually cationic (due to the relative excess of arginine, lysine and/or histidine residues) and amphiphilic, with 30%−50% hydrophobicity (12). They exhibit high structural plasticity and can exist in the form of alpha-helical, beta-sheet, linear extension or mixed a-helical and beta-sheet structures (Figure 1) (13). They have recently shown promise as potential therapeutic agents due to their broad-spectrum antimicrobial properties against a wide array of infection, including drug-resistant bacteria, fungi, acanthamoeba, and viruses, with minimal risk of inducing antimicrobial resistance (11). In principle, HDPs are shown to primarily exert their broad-spectrum and rapid antimicrobial action through three main mechanisms of action, namely the barrel-stave, toroidal pore, and carpet models (Figure 2) (14, 15). The positively charged amino acid residues are responsible for the adsorption of AMPs onto the anionic bacterial membrane (*via* electrostatic interaction) and the hydrophobic residues interact with the lipid tail region of the membrane, culminating in membrane permeation, leakage of fluid into the bacterial cytoplasm and subsequent bacterial cell death (14). In addition to the membrane-targeting action, emerging evidence has highlighted that HDPs can kill microorganisms through several non-membrane perturbing mechanisms, such as biosynthesis of disorganized bacterial membranes and direct intercalation into the membrane, interfering with the intracellular DNA and RNA molecules, and others (7). HDPs are also shown to participate in chemotaxis, immunomodulation, wound healing, anti-biofilm and anti-cancer activities (16–19), offering a wide range of potential therapeutic applications.

The history of HDPs (or AMPs) dates back to 1922 when lysozyme was first discovered in various human tissues and body secretions, including the tear fluids (20). Since then, a wide spectrum of human HDPs have been identified and reported at the OS. These include lactoferrin, alpha- and beta-defensins, cathelicidin (LL-37), ribonuclease, psoriasin and dermcidin, amongst others (9, 21, 22). The expressions and actions of HDPs in several OS diseases have been previously summarized by Kolar and McDermott (23). Since then, there is a growing body of evidence underlining the roles and therapeutic potential of HDPs at the OS, ranging from novel observations at the molecular level (e.g., upregulation of defensins and LL-37 in ocular rosacea) (24) to the advancement of designed HDPs toward human clinical trials (e.g., development of Mel4 as an antimicrobial coating for contact lens) (25). In view of the rapid evolution of this field, this review article aimed to provide an up-to-date, focused review of the spectrum and expression of HDPs at the OS, the roles in major OS diseases, and the therapeutic potential for OS diseases.

## Method of Literature Search

Electronic databases, including MEDLINE and EMBASE, were searched to identify relevant studies on HDPs at the OS. Only English articles were included in this review article. Key words used were “antimicrobial peptide,” “defense peptide,” “ocular surface,” “tear fluid,” “defensins,” “cathelicidin,” “keratitis,” “dry eye,” “atopic keratoconjunctivitis/atopic dermatitis,” “ocular rosacea.” The bibliographies of included articles were manually screened to identify further relevant studies. The final search was last updated in November 2021.

## Spectrum and Characteristics of HDPs at Ocular Surface

A wide array of HDPs have been identified and reported at the OS. In this section, we summarize the sources, characteristics, and functions of important HDPs, including lysozyme, lactoferrin, alpha- and beta-defensins, cathelicidin, ribonucleases, psoriasin, dermcidin, and histatin (Table 1).

**Table 1:** Characteristics and functions of common HDPs at the ocular surface (OS).

**Type**	**Source**	**Functions**
Lysozyme	- Tear fluid (secreted by tubuloacinar cells of lacrimal glands) - Corneal epithelium - Meibomian glands	- Antimicrobial property (*via* hydrolysis and pore formation of cell wall) (20, 26) - Immunomodulatory function *via* interaction with various pattern recognition receptors (26, 27)
Lactoferrin	- Tear fluid (secreted by acinar cells of lacrimal glands) - Conjunctival epithelium - Corneal epithelium - Meibomian glands	- Antimicrobial activity (*via* binding to free iron and membrane permeabilization) (28–30) and anti-biofilm (31) - Immunomodulatory function (anti- and pro-inflammatory) (32, 33) - Antioxidant (*via* inhibition of iron-dependent formation of hydroxyl radicals) (34) - Wound healing (32, 33)
Human alpha-defensins (or HNP)-1 to−4	- Azurophil granules of neutrophils	- All: antimicrobial activity (*via* membrane perturbation) (35) - HNP-1 to−3: immunomodulatory (Pro-inflammatory and anti-inflammatory) (36–38) - HNP-1 to−3: anti-cancer (39, 40)
Human beta-defensins (HBD)-1 to−3	- Conjunctival and corneal epithelium	- All: antimicrobial activity (*via* membrane perturbation) (41, 42) - All: immunomodulatory function (pro-inflammatory and anti-inflammatory) (42, 43) - HBD-3: wound healing (44) - All: anti-cancer (45, 46)
Cathelicidin	- Conjunctival epithelium - Corneal epithelium	- Antimicrobial activity (*via* membrane perturbation) (47–52) and anti-biofilm (47, 53) - Immunomodulatory function (pro-inflammatory and anti-inflammatory) (54, 55) - Wound healing (48, 56) - Anti-cancer (40, 57)
Ribonucleases- (RNases)	- RNase-5: Tear fluid and corneal endothelium - RNase-7: Corneal epithelium and stroma	- Antimicrobial activity (*via* binding to bacterial membrane lipoprotein and membrane perturbation) (58–66) - Immunomodulatory function (activates adaptive immunity) (67, 68) - Angiogenic and neurogenic (69, 70) - Wound healing (71)
Psoriasin	- Conjunctiva - Cornea - Lacrimal gland - Nasolacrimal duct	- Antimicrobial activity (*via* zinc-dependent mechanism) (72, 73) - Immunomodulatory function (chemotaxis, activates adaptive immune system *via* CD4^+^) (74, 75)
Dermcidin	- Corneal epithelium - Tear fluid	- Antimicrobial activity (*via* zinc-dependent mechanism) (76)
Histatin	- Tear fluid	- Antimicrobial activity (*via* membrane perturbation) (77, 78) - Anti-inflammatory function (79) - Wound healing property (80, 81)

*HNP, human neutrophil peptide/human alpha-defensin; HBD, human beta-defensins*.

### Lysozyme

In 1922, lysozyme was discovered by Sir Alexander Fleming during the investigation of his patient with acute coryza. The nasal secretion of the patient was found to completely inhibit the growth of *Micrococcus* spp. (a Gram-positive bacteria). This striking observation prompted a series of experiments, which led to the discovery of lysozyme in various human tissues and body secretions, including tear fluids, saliva, blood, semen, respiratory tract linings, and connective tissues, amongst others (20). Interestingly, the antibacterial potency of lysozyme was influenced by the location of the tissues and types of microbes (e.g., lysozyme in tears was very active against micrococci, but was much less effective against other cocci in other parts of the body), highlighting the specific adaptation of the human immune system against specific pathogens at defined sties (20).

Lysozyme is primarily secreted in the tear fluid by the tubuloacinar cells of the main and accessory lacrimal glands (82) and, to a lesser extent, expressed by corneal epithelium and meibomian glands (83). It constitutes around 20%−30% of the total protein in tear fluids (82). Lysozyme exhibits its broad-spectrum antimicrobial activity *via* dual mechanisms of action (26). First, it hydrolyzes the bacterial cell wall by breaking down the β-1,4 glycosidic linkages between the disaccharides, *N*-acetylmuramic acid (NAM) and *N*-acetylglucosamine (NAG), which forms the backbone of peptidoglycan in the bacterial membrane. Second, the cationic property of lysozyme enables pore formation in the anionic bacterial membrane, which is responsible for its rapid and broad-spectrum antimicrobial activity against a wide range of organisms.

In addition to its antimicrobial activity, lysozyme plays an important immunomodulatory role in host defense. Particularly, it activates lysozyme-dependent degradation of the engulfed bacteria within the phagolysosomes of macrophages and releases pathogen associated molecular patterns (PAMPs) from the lysed bacteria, resulting in a pro-inflammatory response *via* interaction with various pattern recognition receptors (PRRs) such as Toll-like receptors (TLRs), nucleotide-binding oligomerization domain-like receptors (NLRs), and inflammasomes (26). Lysozyme may decrease systemic inflammation by restricting bacterial growth (27). In view of the ubiquitous presence and inherent antimicrobial and immunomodulatory activities of host lysozyme, bacteria have evolved several ingenious resistant mechanisms to survive against lysozyme. These include modification of membrane peptidoglycan, alteration of the membrane charges, and production of protein inhibitors against lysozyme (26). The understanding of the mechanisms of antimicrobial resistance (AMR) related to lysozyme (and potentially other naturally occurring HDPs) is unequivocally pivotal for development of the next generation of synthetic peptide-based therapeutics for tackling AMR.

### Lactoferrin

Lactoferrin, belongs to the transferrin family, is an 80 kDa iron-sequestering HDP. It consists of a polypeptide chain that is folded into two highly symmetrical lobes (N- and C-lobes), which are capable of binding a variety of metal ions including ferric and ferrous ions (28). It is found abundantly in milk and in many other body tissues and secretions, including tears, saliva, sweat, nasal secretion, bronchial mucus, hepatic bile and others (84). Similar to lysozyme, lactoferrin is also primarily synthesized by the acinar cells of the main and accessory lacrimal glands (85). Some evidence has suggested the expression of lactoferrin in meibomian glands (83) and epithelium of conjunctiva and cornea (83, 86). It constitutes around 25% of the total protein in tear fluids, with a concentration of ~2.2 mg/ml (29).

Lactoferrin has been shown to play multi-functional roles in host defense, armed with antimicrobial, anti-biofilm, anti-inflammatory, anti-cancer and anti-complement functions (28, 87). The antimicrobial activity of lactoferrin is attributed to its underlying dual mechanisms of action: (a) binding to free iron, an essential element for microbial growth; and (b) interaction and permeabilization of the anionic bacterial membrane through its positively charged N-terminal, which accounts for its rapid antimicrobial action (28). At the OS, it has been shown to exert broad-spectrum antimicrobial activity against Gram-positive and Gram-negative bacteria, fungi, and viruses (29). It has a strong affinity toward the lipopolysaccharides (LPS) of the Gram-negative bacterial membrane, resulting in increased permeability. Studies have also shown that lysozyme and lactoferrin work in synergy where lactoferrin binds to the lipotechoic acid (LTA) of staphylococcal membrane and enables a greater access of lysozyme to the peptidoglycan (30). Another recent study by Avery et al. (31) showed that lactoferrin exhibits strong antimicrobial and antibiofilm activities against *Acinetobacter baumannii*, which is an important member of the ESKAPE pathogens commonly responsible for multidrug resistance in clinical setting. Interestingly, lactoferrin is ineffective against *Acanthamoeba trophozoites* and this is attributed to the effect of proteases released by *Acanthamoeba* (88).

Lactoferrin has been shown to play an important role in corneal wound closure where it regulates the anti-inflammatory and pro-inflammatory responses (32, 89). Pattamatta et al. (32, 33) demonstrated that lactoferrin stimulates corneal wound healing *via* upregulation of plate-derived growth factor and IL-6, downregulation of IL-1, and reduction of infiltrating inflammatory cells. Lactoferrin also acts as an antioxidant *via* inhibition of iron-dependent formation of hydroxyl radicals, thereby protecting corneal epithelium from oxidation-mediated tissue injury (34). This may have an implication on the pathogenesis of keratoconus (refer to Section Keratoconus). Furthermore, reduced levels of lactoferrin have been associated with systemic mucosal immunity incompetence. Hanstock et al. (90) observed that patients affected by upper respiratory tract infection had a significantly lower concentration of tear lysozyme and/or lactoferrin compared to healthy volunteers, suggesting that lysozyme and lactoferrin may serve as clinically relevant biomarkers for mucosal immune competence.

### Defensins

Defensins are a large family of cysteine-rich HDPs that consist of a predominantly triple-stranded beta-sheet core structure stabilized with three pairs of intramolecular disulfide bridges (91). Depending on the pattern of the disulfide linkage, human defensins can be broadly divided into two groups, namely the alpha- and beta-defensins. Alpha-defensins have a cysteine pairing motif of Cys1–Cys6, Cys2–Cys4, and Cys3–Cys5 whereas beta-defensins form disulfide bridges at Cys1–Cys5, Cys2–Cys4, and Cys3–Cys6 (35, 91). Interestingly, this evolutionarily conserved disulphide bridge motif is similarly observed in defensins found in plants and invertebrates (92, 93).

Human alpha-defensins, also known as human neutrophil peptide (HNP) due to their abundant presence in neutrophils, can be subclassified into 6 main subtypes (HNP-1 to−6). HNP-1 to−4 are found primarily in the azurophil granules of neutrophils (35). HNP-1 to−3 sequences are highly homologous with only difference in a single N-terminal residue; removal of the alanine (the first amino acid of HNP-1 at the N-terminal) gives rise to HNP-2 and substitution of the alanine with aspartic acid yields HNP-3. HNP-5 and−6 are primarily located in the epithelium of Paneth cells of small intestines (35). On the other hand, more than 30 types of human beta-defensins (HBDs) have been described in the literature (94). HBD are mainly synthesized by the epithelial cells, including the conjunctiva, cornea, skin, oral mucosa, lining of respiratory and gastrointestinal tracts, and others (95). As described by McIntosh et al. (96), about 28 novel beta defensins were identified in human genome using the hidden Markov model. Thus far, only few, namely the HBD-1 to−4 and HBD-9 were shown to be involved in host immunity at the OS.

In view of the diverse function of defensins, it is not surprising that a plethora of HNPs and HBDs are abundantly present at a variety of bodily surfaces. At the OS, HNP-1 to−3, but not HNP-4 to−6, have been identified in normal human tears, conjunctival and corneal epithelium, lacrimal gland, and inflamed conjunctiva (in relation to infiltrating polymorphonuclear cells) (22, 97, 98). Similarly, McIntosh et al. (96) discovered an array of HBDs, including HBD-1 to−4, at the corneal and conjunctival epithelium, though the level of HBD-4 was relatively low. Another novel HDP, HBD-9, was discovered at the ocular surface epithelia and corneal stroma by our research group (99, 100). Further studies from our group and others have also shown that the expressions of HBDs are modulated by various PRRs, including TLRs and NLRs (99, 101, 102).

Defensins have been shown to exhibit broad-spectrum antimicrobial activity against bacteria, fungi, enveloped viruses, and parasites (35, 41). Similar to most cationic HDPs, the defensins also perturb the microbial membrane through direct interaction with the anionic and lipid microbial membrane. The antimicrobial efficacy of defensins is likely related to their inherent physicochemical characteristics such as cationicity, hydrophobicity, and amphiphilicity (35). It has been shown that cationicity plays a more important role in Gram-negative infections, whereas increased hydrophobicity enhances the antimicrobial action against Gram-positive infections (103, 104). In addition, synergy between different families of HDPs have been reported; for instance, HBD-2 and LL-37 exhibit synergistic antimicrobial killing of *Staphylococcus aureus*, which is likely accountable for the minimal risk of *S. aureus* infection in inflamed psoriatic skin (105).

**Figure 3:**
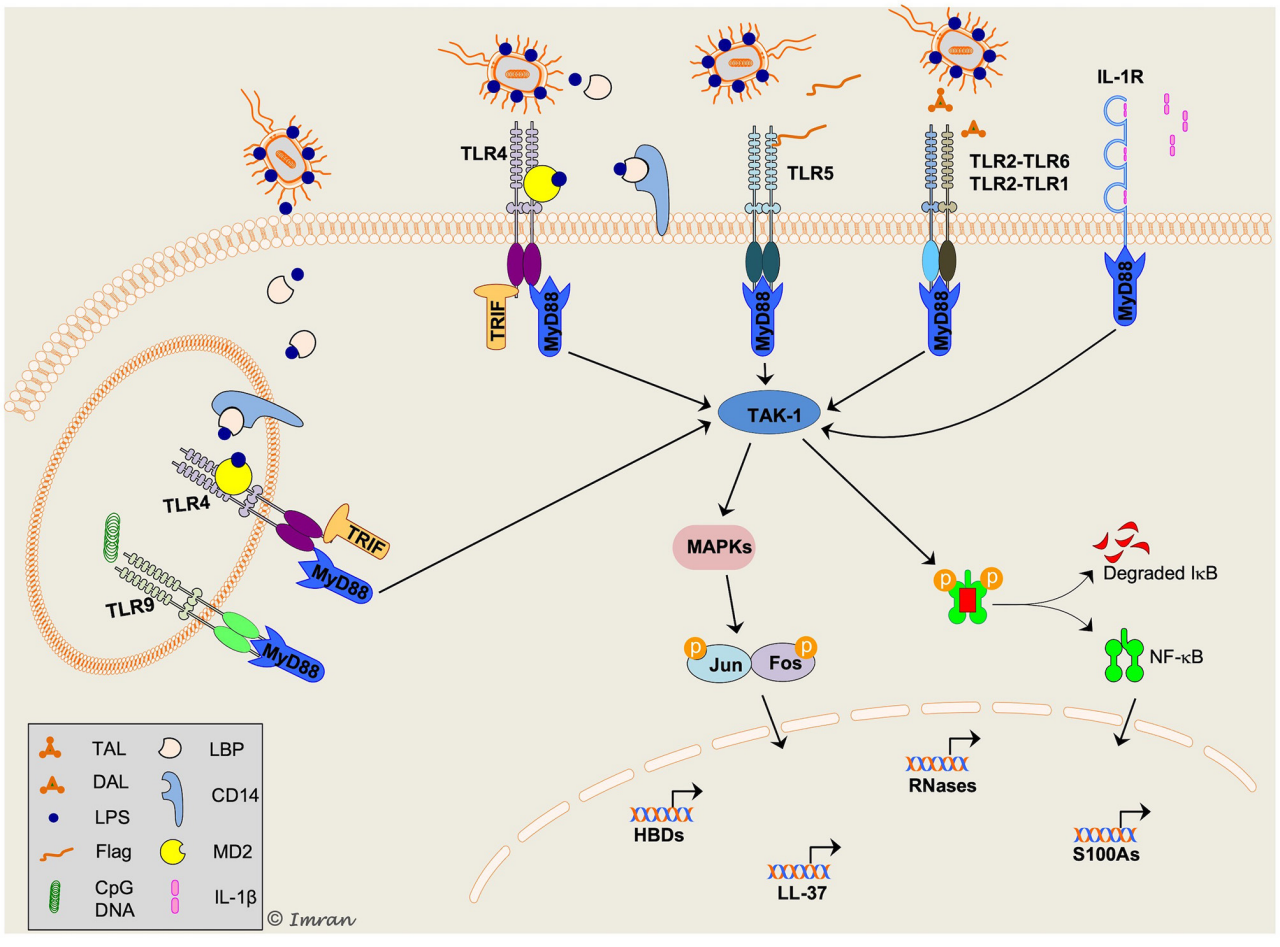
Schematic representation of key signaling mechanisms involved in host defense peptides (HDPs) production in response to bacterial infection. Multiple intracellular signaling pathways are activated downstream of toll-like receptors (TLRs) in response to a variety of pathogen-associated molecular pattern (PAMPs), resulting in production of HDPs and cytokines/chemokines. TLR2/1 and TLR2/6 are shown to recognize diacylated (DAL) and triacylated (TAL) lipopeptides, respectively. TLR4 is present both on cell-surface and intracellularly on endosomes specifically recognizes lipopolysaccharide (LPS). LPS is recognized by LPS binding protein (LBP) and presented to CD14 (present in a soluble form in tear fluid), which transports LPS to myeloid differentiation-2 (MD-2)/TLR4 complex. Flagellin (Flag), a flagellar protein of Gram-negative bacteria, is recognized by TLR5. TLR9 present on endosomes recognizes CpG containing bacterial DNA; however, its role in production of HDPs and associated signaling mechanisms in corneal epithelial cells remain unclear. A pleiotropic cytokine, interleukin-1β (IL-1β) is recognized by IL-1R on cell surface. Activation of toll/IL-1-receptor (TIR) domain of both TLR and IL-1R triggers recruitment of the adaptor molecule myeloid differentiation primary response protein 88 (MyD88). TLR4 signaling can be activated via MyD88 and TIR-domain-containing adaptor protein inducing interferon-β (TRIF). Both MyD88 and TRIF initiate phosphorylation and ubiquitylation of several other molecules (not shown) leading to activation of transforming growth-factor-β activated kinase-1 (TAK1). In the cytosol, TAK-1 triggers activation of mitogen-activated protein kinases (MAPKs) and nuclear-factor-κ-B (NF-κB) pathways. This allows nuclear translocation of NF-κB and activator protein 1 (AP-1; complex of Jun and Fos protein) transcription factors and modulates expression of target HDPs. The scheme was adapted from Mohammed et al. paper (9).

In addition to the antimicrobial function, defensins are endowed with a wide range of functions, including immunomodulatory (pro-inflammatory and anti-inflammatory), wound healing, maintenance of skin barrier, and anti-cancer (Figure 3) (17, 36, 39–41, 43–46, 106). HBD has been shown to orchestrate the cross-talk between innate and adaptive immunity by recruiting T cells and dendritic cells to the infection site through interaction with chemokine (CCR6) receptor (43). HNP-1 regulates inflammation by inhibiting macrophage mRNA translation and secretion of proinflammatory cytokines and nitric oxide, enabling clearance of pathogen and resolution of inflammation with minimal collateral tissue damage (37, 38). Moreover, HBD-3 has been shown to promote wound closure in *S. aureus* infected diabetic wounds (44).

To gain a better understanding of the structure-activity relationship, many research groups have investigated the functional role of the evolutionarily conserved cysteine disulfide bridge moiety of defensins. Although this moiety is widely observed in vertebrate and invertebrate defensins, Wu et al. (42) demonstrated that removal of this structure has no influence on their inherent antibacterial activity against *Escherichia coli*. On the other hand, the chemotactic function (e.g., HBD-3) (42), anti-tumor necrosis factor (TNF)-alpha (e.g., HNP-1) (38), and antiviral activity (e.g., HNP-1 to−3) (107) are abolished when this disulfide moiety is destabilized or removed, suggesting that the disulfide bridges play important immunomodulatory and antiviral roles in innate immunity. These observations provide invaluable insight into the design and development of antimicrobial HDPs that are based on cysteine-rich native templates (108).

### Cathelicidin

Cathelicidins are a large family of AMPs widely found in vertebrates (93, 109). The hallmark of cathelicidin is the presence of highly conserved cathelin domain, which was first identified in pig leukocytes as a cathepsin-L inhibitor and termed “cathelin” based on this property. Cathelicidin proteins comprised of a conserved 14 kDa cathelin domain flanked by a signal peptide (up to 30 residues) on N-terminus and an antimicrobial peptide region on its C-terminus. hCAP18, an 18 kDa preprotein, is the lone member of cathelicidin found in humans (110, 111). Its derivative, hCAP18(104–140), was shown to neutralize lipopolysaccharide (LPS) activity both *in vitro* and *in vivo* (112). Proteinase-3, a proteolytic enzyme in human neutrophils can cleave hCAP18 into an active 37 amino-acid AMP, known as LL-37 (110, 113, 114). Moreover, another serum protease, gastricsin, at low vaginal pH was shown to cleave hCAP18 into a slightly longer active peptide, termed ALL-38 (115).

Since its first discovery in 1988 (116), cathelicidin is the most studied cationic HDP due to its wide-spectrum of activity, including anti-infective, anti-biofilm, anti-cancer, immunomodulatory, chemotactic, and wound healing properties (47, 53, 54, 57, 104, 117–120). The protective function of LL-37 against OS has been widely established (48, 49, 121, 122). LL-37 is constitutively expressed in OS epithelial specimens from healthy living patients and donor cadaveric tissues, including conjunctival and corneal epithelium (96, 123). It has been shown to play an important role in corneal wound healing and protection against various types of microbes at the OS (48, 123). In addition to its antimicrobial activity, Torres-Juarez et al. (55) demonstrated the immunomodulatory effects of LL-37 during mycobacterial infection, including reduction of tumor necrosis factor (TNF)-alpha and IL-17, and promoting the production of transforming growth factor (TGF-beta) and anti-inflammatory IL-10. Furthermore, LL-37 promotes wound healing *via* keratinocyte migration, which occurs *via* epidermal growth factor receptor transactivation (56).

Biochemical studies have elegantly demonstrated that smaller synthetic fragments derived from the parent LL-37 sequence were as effective as the full-length LL-37 (124–128). Studies have revealed that the middle region of LL-37_17−29_ (i.e., FK13) and/or LL-37_18−29_ (i.e., KR12) is largely responsible for the antimicrobial activity of LL-37 and has the ability to form amphipathic helix rich in positive charge, which enables effective interaction with the anionic membrane and subsequent microbial killing (126, 127, 129). In view of its therapeutic promise, a variety of strategies have been adopted to enhance the safety and efficacy of LL-37 and its derivatives (104, 130). Similar to LL-37, its smaller derivatives have shown considerable activity against a range of pathogens, including ESKAPE bacteria, fungi and viruses (47, 50–52). Our group has recently demonstrated that LL-37_17−32_ (FK16 peptide with free N- and C-termini) could also be utilized to improve the activity of conventional antibiotics such as vancomycin against *Pseudomonas aeruginosa*, as a strategy to repurpose the antibiotics and tackle AMR (131).

### Ribonucleases (RNases)

Human ribonucleases (RNases) have an inherent ability to hydrolyze polymeric RNA and share a unique structural similarity to bovine pancreatic RNase A, therefore, also referred to as RNase A superfamily (132, 133). Similar to defensins, members of RNase A superfamily are comprised of six to eight conserved cysteine residues forming disulfide bridges. Genes encoding for human RNases 1 to 13 are clustered on chromosome 14q11.2 (133, 134). RNases are highly cationic and exhibit strong cytotoxic and microbicidal properties. Human RNase-2 (eosinophil derived neurotoxin) and RNase-3 (eosinophil cationic protein) are the first members of RNase A superfamily to show a strong role in host defense against an RNA virus, respiratory syncytial virus (RSV) (58, 59). Further studies have demonstrated that RNase-2 and−3 also have an ability to activate adaptive immunity (67, 68) and possess potent bactericidal and anti-helminthic properties (60–63). RNase-4 and−5 are shown to display potent angiogenic and neurogenic properties (69, 70). RNase-5, also known as angiogenin, has been widely studied due to its immunomodulatory properties. It is shown to be produced by skin keratinocytes and mast cells and has been detected in lacrimal secretions. RNase-5 has been shown to promote corneal endothelial wound healing *via* activation of PI3-kinase/Akt pathway (71), highlighting its therapeutic potential for corneal endothelial diseases. RNase-6 is ubiquitously expressed in immune cells including neutrophils and monocytes. Similar to RNase-3, it also exhibits bactericidal effect through agglutination and membrane disruption (64). Against *Mycobacterium* spp., it has been shown to induce autophagy in the infected macrophages leading to intracellular growth inhibition (135).

RNase-7 and−8 despite being structurally similar, their expression in different bodily tissues is greatly varied. On the OS, RNase-7 is constitutively expressed in healthy corneal epithelium and stroma (65). Further studies have demonstrated elevated levels of RNase-7 in samples collected from patients with bacterial, viral and Acanthamoeba keratitis as well as in CECs treated with cytokines, live bacteria and different pathogenic proteins that activates innate immune receptor signaling (65, 66). Specifically, the signaling mechanisms that are involved in elevation of RNase-7 levels in CECs in response to activation of interleukin 1β (IL-1β)/IL-1 receptor (IL-1R) axis was mapped by our group (65). Notably, the canonical nuclear factor κB (NFκB) transcription factor which mediates transcription of most HDPs in OS epithelium was found to be non-redundant in regulation of RNase-7. It was shown that IL-1b/IL-1R triggered mitogen activated protein kinases (MAPKs) signaling was responsible for RNase-7 regulation in CECs. Further analysis showed that the transcription factors, c-JUN and ATF, are involved in transcription of RNase-7 in CECs. This suggested that a biomarker or protein that directly activates these transcription factors could elicit HDPs in CECs during infection.

### Psoriasin

Psoriasin, or S100A7, represents one of the main members of the S100 family of calcium-binding proteins (136). It is a low molecular weight protein (~11 kDa) which consists of five alpha-helices and the structure relies on the binding of calcium (137). The term “psoriasin” was first coined in 1991 by Madsen et al. (138), who observed the upregulation of this novel HDP in psoriatic skin. Subsequently, its immunomodulatory role in psoriasis was shown to be related to the downstream stimulation of interleukin-1a (IL-1a) expression in human epidermal keratinocytes *via* the receptor for advanced glycation endproducts (RAGE)-p38 MAPK and calpain-1 pathways (139). At the OS, psoriasin was also found to be constitutively present various structures, including the conjunctiva, cornea, lacrimal gland and nasolacrimal ducts (72, 140), highlighting its protective role at the OS.

Psoriasin has been shown to exhibit strong antimicrobial activity against *E. coli* and *S. aureus*, likely *via* a zinc-dependent mechanism (72, 73). The upregulation of psoriasin against *E. coli* was found to be mediated *via* TLR5 (141). Interestingly, studies have shown that the antibacterial efficacy of psoriasin is likely conferred by the central region of the protein (amino acids at 35–80) (73). In addition to its antimicrobial activity, psoriasin has been shown to play essential important immunomodulatory roles, including chemotaxis for CD4^+^ and neutrophils, production of cytokines and chemokines by neutrophils, generation of reactive oxygen species, and release of HNP-1 to−3 (74, 75).

### Dermcidin

Dermcidin (DCD) is an important 110-residue HDP that is constitutively present in the golgi complex and the secretory granules of eccrine sweat. After being proteolytically processed, it is secreted into the sweat and transported onto the epidermal surface of skin as DCD-1L (which constitutes the N-terminal 48 residues of DCD) (76, 142, 143). It has been shown to adopt a unique high-conductance transmembrane hexameric channel architecture comprising trimers of antiparallel helical pairs, which is responsible for its membrane-disruptive antimicrobial mechanism (144).

The presence of DCD was first discovered in 2001 by Schittek et al. (145) and was found to possess broad-spectrum antimicrobial activity that is maintained over a broad pH range and in high salt concentrations, which resembles the human sweat. At the OS, McIntosh et al. (96) observed that dermcidin may be present at the corneal epithelium but this was only detected in one of the nine corneal samples. The presence of dermcidin in tear fluid was further confirmed by You et al. (146). Unlike most HDPs (which are cationic and kill bacteria *via* pore formation), dermcidin is an anionic peptide (147). It exerts its antimicrobial killing through interaction with the anionic bacterial phospholipids with subsequent zinc-dependent formation of oligomeric complexes in the bacterial membrane, resulting in formation of ion channels, membrane depolarization and cell death (76).

### Histatin

Histatin belongs to a family of histidine-rich, cationic HDPs that are produced by the salivary gland into the saliva. They were first identified in 1988 by Oppenheim et al. (77) in human parotid secretion. Within the family, histatin-1,−3 and−5 are the major and most widely studied members and have been shown to exhibit antibacterial, antifungal and wound healing properties (77, 78). Histatin-1 and−3 are encoded by HTN1 and HTN3 genes, respectively, and histatin-5 is a proteolytic product of histatin-3 (148).

The first evidence of the presence of histatin at the ocular surface was demonstrated by Kalmodia et al. (149) in 2019. Histatin-1 was found to be present in normal human tears and reduced in aqueous-deficient dry eye disease by around 10-fold, suggesting the potential diagnostic value in evaluating dry eye disease. Based on *in vitro* studies, histatin-1 has been shown to enhance human corneal epithelial wound healing (80). In addition, histatin-1 can significantly reduce lipopolysaccharide-induced inflammatory signaling and production of nitric oxide and inflammatory cytokines *via* the JNK and NF-kB pathways in RAW264.7 macrophages (79). The multi-faceted properties of histatin, including antimicrobial, anti-inflammatory and wound healing properties, are particularly attractive for ocular surface diseases, especially infectious keratitis where inflammation overdrive and persistent epithelial wound are common sequelae of the infection (81, 150, 151).

## Roles of HDPs in Major Ocular Surface Diseases

It is evident that HDPs play important roles in innate immunity and crosstalk between innate and adaptive immunity. In this section, we aim to provide a concise overview of the roles of HDPs in major OS diseases.

### Infectious Keratitis

Infectious keratitis (IK) represents a major cause of corneal blindness worldwide (152). It has been estimated to cause 1.5–2 million new cases of monocular blindness every year, highlighting its significant burden on human health, healthcare resources and economy (152–154). Subject to geographical, temporal and seasonal variations, bacteria and fungi are the most common culprits for IK globally (150, 155–161). Broad-spectrum topical antimicrobials are currently the mainstay of treatment for IK but adjuvant therapy such as amniotic membrane transplant, therapeutic corneal cross-linking treatment (i.e., PACK-CXL) and therapeutic keratoplasty are often required to manage refractory cases of IK (162–166).

The pivotal roles of HDPs in IK are supported by a number of *in vitro* and *in vivo* observations and experiments (9). McIntosh et al. (96) investigated differential gene expression of HDPs in non-infected and infected eyes and demonstrated that some HDPs, notably HBD-3 and LL-37, were significantly elevated during OS infection. In addition, HBD-2 and−3, LL-37, MIP-3α, and thymosin β-4 were shown to exhibit moderate to strong *in vitro* antimicrobial activity against a range of ocular pathogens, including *S. aureus, P. aeruginosa*, adenovirus and HSV-1 (49, 123). Furthermore, cathelicidin-deficient/knockout mice were found to be more susceptible to *P. aeruginosa* corneal infection when compared to the wild type mice, underlining the antimicrobial function of cathelicidin at the OS (122). Synergistic antimicrobial action among different HDPs has also been reported in several studies (167, 168). For instance, Chen et al. (167) demonstrated that various combinations of HDPs, including HBD-1 to−3, LL-37 and lysozyme, exhibited synergistic or additive antimicrobial effect against *S. aureus* and *E. coli*.

The role of HDPs has also been implicated in other types of IK such as fungal and Acanthamoeba keratitis (9). Our recent study demonstrated that a range of HDPs, including HBD-1,−2,−3 and−9, LL-37 and S100A7, were upregulated during the active phase of fungal keratitis and returned to the baseline level upon resolution of the infection (169). Interestingly, there was a preferential increase in mRNA expression of different types of HDPs, with HBD-1 and−2 being most commonly upregulated (90% of the cases) and LL-37 being least commonly upregulated (35% of the cases), highlighting the pathogen-specificity of HDPs. Similarly in Acanthamoeba keratitis, a wide range of HDPs such as HBD-2 and−3, LL-37, LEAP-1 and−2, and RNase-7 (but not HBD-1), were shown to be upregulated (66). Interestingly, HBD-1 and HBD-9 were significantly downregulated in Acanthamoeba keratitis (66, 170). Taken together, it is evident that HDPs serve as an integral component of the innate immunity of the OS, *via* their broad-spectrum and rapid antimicrobial activity against a wide range of ocular pathogens. These unique characteristics also render HDPs (usually those that are membrane-active) an attractive class of antimicrobial agent for managing IK, particularly in the face of polymicrobial keratitis and emerging antimicrobial resistance (152, 158, 171, 172).

### Dry Eye Disease and Sjogren's Syndrome

Dry eye disease (DED) is one of the most common ocular surface morbidities with severe impact on vision and quality of life of affected individuals (173, 174). According to the recent TFOS DEWS II report, DED is defined as “a multifactorial disease of the ocular surface characterized by a loss of homeostasis of the tear film, and accompanied by ocular symptoms, in which tear film instability and hyperosmolarity, ocular surface inflammation and damage, and neurosensory abnormalities play etiological roles” (173). Sjogren's syndrome is a systemic autoimmune disease that primarily affects the lacrimal and salivary exocrine glands, leading to dry eyes and dry mouth (175). It is caused by lymphocytic infiltration of the exocrine glands secondary to the abnormal B- and T-cell autoimmune response against the auto-antigens, particularly SSA and SSB (175).

Several studies have demonstrated the dysregulation of HDPs in the DED. A range of HDPs, particularly lysozyme and/or lactoferrin, have been shown to be reduced in various types of DED, including SS and non-SS-related DED (176), evaporative DED (177), graft versus host disease (GvHD)-related DED (178), and others. Furthermore, HBD-2 and HBD-9 are found to be upregulated and downregulated, respectively, whereas HBD-1 and−3 remain unchanged in DED (100, 179). In addition, tear HDPs may also serve as useful biomarkers in DED. Studies have shown that tear lactoferrin was significantly reduced in various types of DED, including SS-related and non-SS-related DED, Steven–Johnson syndrome and evaporative DED secondary to meibomian gland dysfunction (177, 180). Sonobe et al. (176) recently demonstrated an inverse correlation between reduced lactoferrin concentration in tears and increased severity of DED using a novel and innovative microfluidic paper-based analytical device (μPAD). It has been shown that reduced level of lactoferrin serves as a good biomarker for distinguishing SS-related DED from non-SS-related DED (181), and for diagnosing DED in postmenopausal patients (182). The reduction of these tear HDPs in DED, in addition to the breakdown of corneal epithelium and increased bacterial load associated with DED, may potentially account for the increased risk of IK (though lack of strong evidence) (183, 184).

### Keratoconus

Keratoconus is a bilateral, non-inflammatory corneal condition characterized by progressive corneal thinning and protrusion with resultant myopia and irregular astigmatism. It is the most common corneal ectatic disorder with an estimated prevalence of 1:2,000 to 1:400 people (185, 186). Depending on the severity and stability of keratoconus, it can be managed with glasses, soft and rigid contact lens, corneal cross-linking, intrastromal corneal ring segments, and corneal transplantation if all other measures fail (187–189). Although uncommon, keratoconus still remains a leading indication for corneal transplantation in many countries (190, 191).

The pathogenesis of keratoconus is likely to be multifactorial, contributed by genetic predisposition, environmental factors, proteolytic degradation of collagen, and mechanical trauma such as eye rubbing (192). Several molecular and proteomics studies (193–196) have also demonstrated the upregulation of certain tear proteins and inflammatory molecules in keratoconus, including interleukin-6, TNF-alpha, matrix metalloproteinases (MMP)-1,−3,−7,−9, and−13, lipocalin-1, neutrophil-defensin 1 precursor, mammaglobulin-B precursor, and keratin types 1 and 2, suggesting that inflammation plays a role in the pathogenesis of keratoconus. A recent proteomic study by Yam et al. (197) demonstrated that the epithelial and stromal proteins in keratoconic corneas were altered. The proteomic changes were primarily related to developmental and metabolic disorders (particularly in relation to mitochondria), cellular assembly, tissue organization and connective tissue disorders (particularly in relation to endoplasmic reticulum protein folding). Interestingly, the changes were not limited to the “cone area” but also involved the peripheral non-cone region of the keratoconic corneas. In addition, patients with keratoconus were found to have a significantly lower level of tear lactoferrin and the amount of reduction correlated with the severity of keratoconus (198). It is postulated that reduced lactoferrin results in accumulation of free iron in the tear fluids and iron deposition on the cornea (“Fleischer's ring”), thereby increasing cytotoxicity to the corneal epithelial cells (199). Based on these observations, Pastori et al. (199) have demonstrated that the oxidative stress induced by the tears in keratoconic patients, due to increased free iron, may be dampened by lactoferrin-loaded contact lens, potentially deterring the progression of keratoconus.

### Pterygium

Pterygium is a common inflammatory ocular surface disease that is commonly encountered in tropical countries, with an estimated prevalence of 12% (200). It is characterized by fibrovascular growth of the conjunctiva into the cornea, resulting in ocular surface discomfort, pain, visual disturbance and impairment (if visual axis is encroached upon) (201). The pathogenesis of pterygium is likely attributed to a number of factors, including chronic ultraviolet radiation, human papillomavirus infection, oxidative stress, and genetic predisposition (202). So far, few groups have examined the role of HDPs in patients with pterygium. Ikeda et al. (98) observed the presence of HBD-2 in one of two conjunctival tissues of pterygium but in none of all eight normal conjunctival samples. In addition, Zhou et al. (203) reported an upregulated expression of HNP-1 to−3, and calcium-binding proteins S100A8 and S100A9 in the tear fluids of eyes affected with pterygium. Another demonstrated the upregulation of HBD-1 and−2 along with a downregulation of HBD-9 in pterygium (204). These observations may be related to the underlying fibrovascular proliferative changes or inflammation. It was also suggested that the dysregulation of these HDPs may play an important contributory role to the pathogenesis of pterygium and may serve as useful biomarkers for predicting the recurrence of pterygium (203).

### Post-ocular Surface Surgery and Wound Healing

The integrity of corneal epithelium is of utmost importance for ocular surface defense. Corneal wound healing relies on the regenerative capability of limbal stem cells and involves a complex process of cell death, migration, proliferation, differentiation, and remodeling of extracellular matrix (205). The integral role of HDPs for ocular surface wound healing has been evidently demonstrated in many studies. Zhou et al. (206) observed that the level of HNP-1 to−3 in tear fluids increased significantly after surgical removal of ocular surface neoplasm and returned to the baseline level after complete healing. Moreover, the concentration of HNP-1 and−2 reached a therapeutic level at day 3 postoperative (206). In addition, upregulation of HBD-2 mRNA expression was observed during the phase of corneal re-epithelialization (207).

Similarly, Huang et al. (48) previously demonstrated that LL-37 was increased in injured corneal epithelial cells (CEC), and recombinant LL-37 was capable of increasing the pro-inflammatory cytokines from CECs through the activation of G-protein coupled receptors (i.e., formyl peptide receptor-like 1). Application of vitamin D on wounded mouse corneas was shown to delay the normal wound healing process and increase the production of cathelin-related antimicrobial peptide (CRAMP) (208). However, the cause-effect relationship between CRAMP and corneal wound healing remains unknown but it was suggested that the increase levels of HDPs during epithelial defect would protect the cornea from infection during the healing phase. Recent studies have demonstrated that a deficiency of vitamin D receptors significantly delays the corneal wound healing and decreases the nerve density (209, 210). These findings suggest that HDPs play a crucial role in wound healing and protection against ocular surface infection.

### Atopic Dermatitis and Allergic Keratoconjunctivitis

Atopic dermatitis (AD) is the most common inflammatory skin condition characterized by intense pruritus and chronic, relapsing eczematous lesions (211). The lifetime prevalence has been estimated at 20% (211). The pathogenesis of AD is multifactorial, with loss-of-function of the filaggrin gene (which regulates the epidermal barrier function), overgrowth of *S. aureus* (which may be caused by the dysregulation of HBD), IgE-mediated sensitization, and neuroinflammation playing important contributory roles (105, 211). Patients with AD may also suffer concurrently from atopic keratoconjunctivitis (AKC), which is a potentially sight-threatening ocular surface disease. Vernal keratoconjunctivitis (VKC) is another severe form of allergic eye disease that primarily affects the children and young adults (212).

Several studies have implicated the roles of HDPs in AD, AKC and VKC. Both HBD-2 and LL-37 are known to possess good antimicrobial activity against *S. aureus* and they work in synergy (105). Patients with AD are found to have substantially lower expressions of HBD-2 and−3, LL-37, and dermcidin, which may explain their increased susceptibility to staphylococcal skin infection compared to patients with psoriasis (105, 213). Similarly, patients with AKC are at risk of developing staphylococcal and herpetic infectious keratitis (214), which may be linked to the downregulation of mBD-2 mRNA at the ocular surface based on *in vivo* murine allergic eye conjunctivitis studies (215). Hida et al. (216) observed significantly higher levels of HNP-1 to−3 in the tears of patients with AKC complicated by allergic corneal epithelial disease compared to healthy patients or AKC patients with no corneal disease, suggesting a potentially protective role of HDP in corneal complications related to allergic eye disease. In addition, tear lactoferrin is reduced in VKC and the underlying mechanism is likely not related to lacrimal gland dysfunction but other factors since the level of tear lysozyme is unaffected (217).

### Rosacea

Rosacea is a chronic, relapsing inflammatory skin disease that affects around 5% of the population (218). The risk of ocular surface involvement may develop in up to 70% of the rosacea patients and may occur with or without concurrent facial/skin rosacea (3). It can result in a wide array of ocular symptoms and signs, ranging from grittiness, visual blurring, and pain to sight-threatening complications such as corneal infection and perforation. The pathogenesis of rosacea remains to be fully elucidated; however dysregulation of the innate immunity (e.g., dysfunctional expression of HDPs) has been implicated, in addition to a number of environmental factors, genetic predisposition, and neurovascular dysregulation (219). The level of LL-37 is significantly increased in the skin epidermis in rosacea, which promotes skin inflammation *via* leukocyte chemotaxis and angiogenesis (220, 221). Gokcinar et al. (24) recently examined the role of HDPs in ocular rosacea and observed that the gene expressions of a wide range of HDPs, including tear HNP-1 to−3 and HBD-2, and conjunctival LL-37, were upregulated. On the other hand, tear lactoferrin was found to be reduced in rosacea (222).

## Therapeutic Potentials of HDPs for Ocular Surface Diseases

Despite their promising potential as effective antimicrobial and immunomodulatory therapies, several issues have impeded the successful translation of HDPs into clinical use. Complex structure-activity relationship, susceptibility to host/bacterial proteases and physiological conditions, pro-inflammatory properties, discrepancy between *in vitro* and *in vivo* efficacies, and toxicity to the host tissues are the main barriers (14, 23, 223, 224)Furthermore the lack of interest and investment from the pharmaceutical companies, stemming from limited life-span of antimicrobial therapy and low profits, poses another significant hurdle for the development of new antimicrobial agents (225). Herein, we present some of the key HDP-based molecules that have completed *in vivo* animal studies and are in the developmental pipeline for treating ocular surface diseases. These include B2088 branched peptide, Esculentin1–21(NH2), RP444, melimine/Mel4 antimicrobial coating for contact lens, epsilon-lysylated melittin (MEL-4), CaD23, histatin-5, and endogenous LL-37 (Table 2).

**Table 2:** A summary of host defense peptide (HDP)-based molecules that are in the development pipeline for ocular surface diseases.

**Molecules** **(sequence)**	**Primary sources**	**Current development stage**	**Activities**
B2088^*^ ([RGRKVVRR]_2_KK)	HBD-3 (C-terminal)	Pre-clinical stage	- Good activity against PA (108, 226) - Synergism with gatifloxacin and tobramycin (108)
Esculentin1–21(NH2) (GIFSK LAGKK IKNLL ISGLK G-NH_2_)	Esculentin (N-terminal)	Pre-clinical stage	- Good activity against SA and PA (227, 228) - Good anti-biofilm activity against PA (228, 229)
RP444 (FAOOF AOOFO OFAOO FAOFA FAF)	Cecropin and magainins	Pre-clinical stage	- Good activity against Gram-positive and Gram-negative bacteria (230)
Melimine/Mel4 (KNKRK RRRRR RGGRR RR)	Melittin and protamine	Pre-clinical stage + phase 3	- Good activity against Gram-positive and Gram-negative bacteria (231, 232) - Reduces risk of CL-related infection (if CL coated with Mel4) (233)
MEL-4^**^ (GIGAV L*K*VLT TGLPA LISWI *K*RKRQ Q)	Melittin (full-length)	Pre-clinical stage	- Good activity against Gram-positive and Gram-negative bacteria and fungi (234)
CaD23 (KRIVQ RIKDW LRKLC KKW)	Cathelicidin and HBD-2	Pre-clinical stage	- Good activity against SA, MRSA and PA (104) - Strong additive effect when used with levofloxacin or amikacin (235)
Histatin-5 (DSHAK RHHGY KRKFH EKHHS HRGY)	Histatin-5	Pre-clinical stage	- Promote corneal wound healing (81)

*HBD, human beta-defensin; SA, Staphylococcus aureus; MRSA, methicillin-resistant S. aureus; PA, Pseudomonas aeruginosa; CL, contact lens*.

* *This is a branched peptide. The duplicating residues are in bracket*.

** *The italicized “K” residue refers to epsilon-lysylated lysine residue. This MEL-4 molecule is different from the other Mel4 molecule*.

### B2088 Branched Peptide

B2088 is a covalent dimeric peptide that is derived from the C-terminal of HBD-3 [peptide sequence: (RGRKVVRR)_2_KK] (226). The development of this branched peptide was started in 2007 where Liu et al. (236) demonstrated that the linear form of HBD3 maintained similar antimicrobial efficacy and exhibited lower cytotoxicity and haemolytic activity compared to the native form of HBD3, after refining the hydrophobicity and substituting the cysteine residues with various amino acids. Such properties were postulated to be related to the removal of the disulfide bridges and the loss of secondary structure. Bai et al. (237) further enhanced the antimicrobial activity and reduced the host toxicity of linear HBD3 analogs by shortening the HBD3 to 10 amino acids from the C-terminal end. Taking it further, the antimicrobial efficacy of the truncated HBD3 was further optimized *via* dimerization at the lysine, which yielded the final lead compound of B2088 (108, 226).

B2088 has been shown to demonstrate strong antimicrobial activity against Gram-negative bacteria, particularly *P. aeruginosa* (108, 226). It exerts its bacterial killing through the binding of lipid A and disruption of supramolecular organization of lipopolysaccharides, a major component of the outer membrane of Gram-negative bacteria. In addition, B2088 strong synergism with various antibiotics through time-kill and checkerboard assays. This was further validated in an *in vivo* murine *P. aeruginosa* keratitis study where B2088 0.05%-gatifloxacin 0.15% combination treatment reduced the bacterial burden of corneal infection by an additional 1 LogCFU compared to gatifloxacin 0.3% alone (108).

### Esculentin-1a(1–21)NH_2_

The skin of amphibians contains a rich source of HDPs (238). Esculentin-1a is a type of frog-derived HDP isolated from the skin of *Rana esculenta*, or now known as *Pelophylax lessonae/ridibundus*. The modified version, Esculentin-1a(1–21)NH_2_, is composed of the first 20 amino acids of esculentin-1a with a glycinamide residue at the C-terminal end (peptide sequence: GIFSKLAGKKIKNLLISGLKG-NH_2_) (227). It has been shown to demonstrate strong *in vitro* antimicrobial activity against various *P. aeruginosa* laboratory strains (both invasive and cytotoxic strains) and clinical strains (isolated from eyes with keratitis and conjunctivitis), and *Staphylococci species* (with a MIC range of 1–16 μM) (227). In an *in vivo* murine bacterial IK model infected with cytotoxic *P. aeruginosa* strain, topical treatment of esculentin-1a(1–21)NH_2_ significantly reduces the bacterial load, clinical severity and recruitment of inflammatory cells to the infected corneas measured by the relative myeloperoxidase activity (227). In addition, it was shown to exhibit anti-biofilm activity against *P. aeruginosa* (228, 229) and prolong the survival of PAO1-infected mice in both sepsis and pneumonia models (228). The potent activity against both planktonic and sessile forms of *P. aeruginosa* was ascribed to its underlying membrane perturbation activity (228).

### RP444

The development of RP444 was inspired by the “freedom from infection” observed in Cecropia moth and African clawed frog, which is attributed to the cecropins and magainins peptides, respectively (113). RP444 is a 23-amino acid designed HDP primarily composed of phenylalanine, alanine and ornithine, which is an unnatural amino acid used to replace lysine residue to enhance antimicrobial activity and proteolytic stability (peptide sequence: FAOOFAOOFOOFAOOFAOFAFAF) (230). This designed HDP possesses a broad-spectrum antimicrobial activity against a range of Gram-positive and Gram-negative bacteria (MIC ranges between 4 and 64 μg/ml) and anti-biofilm efficacy. Similar to other natural and synthetic HDPs, RP444 exhibits rapid bacterial killing within 30–60 min with no risk of developing resistance. Further *in vivo* murine bacterial keratitis study showed that RP444 was able to significantly reduce the bacterial load and clinical severity of *P. aeruginosa* keratitis and reduce inflammatory cell infiltration toward the infected site (230).

### Melimine and Mel4 Antimicrobial Coating for Contact Lenses

Melimine is a 29-amino acid cationic synthetic HDP derived from melittin (from honeybee venom) and protamine (from salmon sperm) (239). This hybrid HDP combines the C-terminals of both melittin and protamine, yielding a total cationic charge of +14 (peptide sequence: TLISWIKNKRKQRPRVSRRRRRRGGRRRR). When attached to contact lenses, either through adsorption or covalent binding, melimine demonstrates higher antimicrobial activities against both Gram-positive and Gram-negative bacteria than melittin or protamine alone (239). In addition, the hemolytic activity of melimine is significantly lower than melittin. Furthermore, *in vivo* rabbit models successfully showed that melimine-coated lenses were safe to wear and they prevented bacterial growth on contact lenses, which consequently reduced the rate and severity of adverse reactions such as contact lens-induced acute red eye (CLARE), contact lens-induced peripheral ulcers (CLPUs) and IK (240–242). This suggests that hybridization of two different HDPs serves as a novel strategy to enhance antimicrobial efficacy and reduce toxicity.

However, when the melimine-coated contact lenses were tested in a human clinical trial, these lenses were paradoxically associated with significantly higher corneal staining compared to uncoated lenses at day 1 (241). To overcome this unforeseen corneal toxicity, the same research group has further refined the hybrid HDP, which has led to the creation of Mel4 – a truncated version of melimine with +14 net charge (peptide sequence: KNKRKRRRRRRGGRRRR) (231). This modified HDP exhibits modest antimicrobial activity against a broad range of Gram-positive and Gram-negative bacteria, with good *in vivo* safety demonstrated in rabbit and human trials (231). The mechanism of action of Mel4 against *P. aeruginosa* was found to be related to the neutralization of lipopolysaccharide and disruption of cytoplasmic membrane whereas its action against *S. aureus* was likely attributed to the release of autolysins with resultant cell death instead of pore formation (232, 243). A recent randomized controlled trial demonstrated that Mel4-coated antimicrobial contact lens was able to reduce corneal infiltrative events by at least 50% when compared to uncoated control lens during extended wear over 3 months (233).

### Epsilon Lysylated Melittin (MEL-4)

Being as one of the main basic and cationic amino acids, lysine serves as a major constituent of many naturally occurring and synthetic HDPs (244, 245). In addition to the l- and d-form, lysine can also exist in epsilon form (ε-) where the NH_2_ group at the side chain of L-lysine is linked to the alpha-carbon. ε-Poly-l-lysine (EPL) is a basic polyamide consisting of 25–30 ε-lysine that is naturally produced by *Streptomycetaceae* and *Ergot* fungi (246). It is commonly used as a food preservative with strong antimicrobial activity (247, 248). Compared to alpha-poly-l-lysine, EPL exhibits enhanced antimicrobial efficacy against a range of Gram-positive and Gram-negative bacteria (248, 249). Employing the similar strategy, Mayandi et al. (234) explored the selective incorporation of ε-lysine in melittin, which is a potent yet toxic HDP that is found in honeybee venom. They showed that ε-lysylation of melittin, in particular MEL-4 (different from the Mel4 described in the above Pterygium Section), improved the cell selectivity of the synthetic HDP toward a range of Gram-positive and Gram-negative bacteria with reduced host cytotoxic and hemolytic activities, whilst maintaining the *in vivo* efficacy of melittin (234). This suggests that ε-lysylation may serve as a novel strategy for improving the cell selectivity in lysine-rich HDPs.

### Hybridized LL-37 and HBD-2 (CaD23)

LL-37 and HBDs are major groups of HDP that have been shown to play vital roles in various ocular surface diseases, particularly infectious keratitis. Inspired by these observations, our group recently developed a novel molecule, CaD23, *via* rationale hybridization of LL-37 and HBD-2 (peptide sequence: KRIVQRIKDWLRKLCKKW), and demonstrated good antimicrobial activity against a range of organisms commonly responsible for infectious keratitis, including *S. aureus*, MRSA and *P. aeruginosa* (104). The therapeutic potential of CaD23 was further substantiated by the strong *in vivo* antimicrobial activity against *S. aureus* in a pre-clinical murine model with good safety profile.

In addition, CaD23 demonstrates eight times faster antimicrobial action when compared to amikacin, a commonly used antibiotics for infectious keratitis (104). CaD23 also demonstrates a strong additive effect when used in combination with amikacin and levofloxacin against *S. aureus* and MRSA, underscoring the translational potential of peptide-antibiotic combined therapy in clinic (235). More importantly, when *S. aureus* was exposed to 10 consecutive sub-lethal concentration of treatment, the bacteria did not develop any antimicrobial resistance against CaD23 whereas it developed significant antimicrobial resistance against amikacin by 32-fold (104). The rapid antimicrobial action (thence low risk of AMR) is likely attributed to its membrane-permeabilizing properties, evidenced by a combination of experimental and computational studies. Moreover, the molecular dynamics (MD) simulations study revealed the importance of alpha-helicity, cationicity, hydrophobicity and amphiphilicity in contributing to the antimicrobial action of CaD23 (235).

### Histatin-5

Histatin peptides have been shown to demonstrate antimicrobial activity and wound healing properties. Based on a combination of *in vitro* and *in vivo* murine studies, Shah et al. (81) demonstrated that histatin-5 was able to promote corneal wound healing, and the effect pro-migratory effect was extracellular signal-regulated kinase 1/2 (ERK1/2) dependent. The authors were also able to determine that the C-terminal of histatin-5 (i.e., SHRGY) was the critical functional domain responsible for the wound healing property. These findings highlighting the potential of histatin-5 and/or the SHRGY pentapeptide for further development into clinical therapeutics for ocular surface diseases such as neurotrophic keratopathy or persistent corneal epithelial defect following infection or injury.

### Endogenous LL-37 for Atopic Dermatitis

Understanding of the dysregulated expression of HDPs provides a unique opportunity to explore new therapeutic avenue in managing atopic dermatitis and potentially allergic eye diseases. As mentioned, a number of HDPs, including defensins and LL-37, are downregulated in the AD skin lesions (250). It has also been shown that the expression of LL-37 at the skin can be induced by the active 1,25 dihydroxy-vitamin D, which is regulated by the TLR-2 in keratinocytes and monocytes (251). In addition, the severity of AD is inversely proportionate to the level of LL-37 (252). Leveraging on these observations, several research groups have investigated and demonstrated that administration of oral vitamin D may improve the clinical severity of AD (253, 254), accompanied by an increased level of LL-37 (252). Similar strategy can potentially be employed for treating OS diseases, including allergic eye disease.

## Future Directions

Currently there are a few clinical trials underway investigating the potential translation of HDPs from bench to clinics. Learning from the previous experience of other trials, particularly those that had reached but failed phase 3 trials (255, 256), it is important to select clinical areas and diseases that are likely to benefit from HDP treatment; for instance, comparing the efficacy between HDPs and antibiotic treatment for diseases caused by multi-drug resistant infection instead of routine and mild infection (which can be simply managed by current antibiotic treatment) is more likely to yield significant and clinically relevant results (130). In addition, based on the synergistic effect and benefit of reducing dose-related toxicity and AMR, researchers are exploring the use of HDP as adjuvant therapy in addition to antibiotic instead of monotherapy (108, 235). Furthermore, the increasingly recognized multi-faceted biological functions of HDPs, including anti-biofilm, immunomodulatory, wound healing, and anti-cancer properties, have yet to be fully capitalized in the clinic. For instance, HDPs such as defensins and lactoferricin have been shown to exert strong anti-cancer activity against various types of cancer, including colorectal, bladder, neuroblastoma, melanoma, and cutaneous squamous cell carcinoma (257). Nonetheless, the effect of HDP on OS neoplasia (e.g., squamous cell papilloma / carcinoma) has never been investigated or reported, highlighting a potential area for future research.

As there is no one set rule or principle to predict the efficacy and toxicity of designed HDPs, the infinite chemical space renders the design of HDPs a formidable task (7). With the rapid advancement in bioinformatics study (including molecular dynamic simulation), artificial intelligence and drug delivery technologies, efficient design and development of more effective HDPs are more likely to be achieved (130, 258). Integrating synthetic HDPs with novel delivery systems (e.g., nanoparticles, liposomes) may serve as a useful strategy to enhance the proteolytic stability and reduce toxicity of HDPs in the future (130, 259). Stimulation of the production of endogenous HDP using FDA-approved drugs or supplements, for instance using 4-phenylbutyrate and/or vitamin D to increase the level of LL-37, may also serve as a useful strategy in exploiting the benefits of HDP (251, 260, 261). Such an approach helps overcome the significant hurdles encountered during the bench-to-bedside translational process, including the regulatory barriers, for synthetic HDP-based molecules. In addition, the advancement in proteomics and whole genome sequencing technologies could facilitate the mining of previously unknown and undetected natural gene-encoded HDP sequences (262, 263), which can be utilized for therapeutic use in the future.

## Author Contributions

Study design and conceptualization: DT. Literature review, data collection, and manuscript drafting: DT and IM. Data interpretation: DT, IM, RL, RB, and HD. Critical revision of manuscript: RL, RB, and HD. Approval of the final version of manuscript. All authors contributed to the article and approved the submitted version.

## Funding

DT is supported by the Medical Research Council/Fight for Sight (FFS) Clinical Research Fellowship (MR/T001674/1), the FFS/John Lee, Royal College of Ophthalmologists Primer Fellowship (24CO4), and the University of Nottingham International Research Collaboration Fund (A2RRG1). IM acknowledges funding support from the Medical Research Council – Confidence in Concept Scheme (MRC-CIC_2019-028).

## Conflict of Interest

The authors declare that the research was conducted in the absence of any commercial or financial relationships that could be construed as a potential conflict of interest. The reviewer HO declared a shared parent affiliation with the authors RL and RB to the handling editor at the time of review.

## Publisher's Note

All claims expressed in this article are solely those of the authors and do not necessarily represent those of their affiliated organizations, or those of the publisher, the editors and the reviewers. Any product that may be evaluated in this article, or claim that may be made by its manufacturer, is not guaranteed or endorsed by the publisher.
